# Renal outcomes associated with sodium–glucose cotransporter 2 inhibitor use in kidney transplant recipients with type 2 or posttransplant diabetes: A propensity score overlap weighting retrospective cohort study

**DOI:** 10.1097/MD.0000000000050385

**Published:** 2026-09-04

**Authors:** Guillermo Edinson Guzmán, Fabio Figueroa, Santiago Granados, Oriana Arias-Valderrama, Juan Lukas Ordóñez, Carlos Eduardo Durán

**Affiliations:** a Departamento de Endocrinología, Fundación Valle del Lili, Cali, Colombia; b Facultad de Ciencias de la Salud, Universidad Icesi, Cali, Colombia; c Centro de Investigaciones Clínicas, Fundación Valle del Lili, Cali, Colombia; d Departamento de Nefrologia, Fundación Valle del Lili, Cali, Colombia.

**Keywords:** graft survival, kidney transplantation, posttransplant diabetes mellitus, sodium–glucose cotransporter 2 inhibitors, type 2 diabetes mellitus

## Abstract

Posttransplant diabetes mellitus (PTDM) increases the risk of graft failure and mortality. Sodium–glucose cotransporter 2 inhibitors (SGLT2i) improve cardiovascular and renal outcomes in type 2 diabetes mellitus (T2DM), but evidence in kidney transplant recipients (KTRs) with T2DM or PTDM remains limited. We conducted a retrospective cohort study including adult KTRs with pretransplant T2DM or PTDM. Exposure was defined as continuous SGLT2i use for ≥90 days. The primary outcome was a composite of graft loss, all-cause mortality, or a ≥2-fold increase in serum creatinine relative to the week-4 posttransplant value occurring >3 months after baseline. Secondary outcomes included longitudinal changes in HbA1c, estimated glomerular filtration rate (eGFR), and urine protein-to-creatinine ratio (UPCR), as well as adverse events (acute kidney injury [AKI] and urinary/genital infections). Propensity score overlap weighting was used to balance baseline covariates, and time-to-event analyses were performed using an overlap-weighted Cox regression model. A total of 185 patients were included (116 SGLT2i users and 69 non-users), with a mean follow-up of 121 months. The primary composite outcome occurred less frequently in the SGLT2i group (*P* < .001). In an overlap-weighted Cox regression model, SGLT2i use was associated with a lower hazard of the composite endpoint (HR 0.30, 95% CI: 0.17–0.55; *P* < .001). Safety outcomes did not differ significantly between groups, including urinary sepsis/hospitalization categories, ketoacidosis, and genital infections. Among KTRs with T2DM or PTDM, SGLT2i use was associated with a lower combined risk of graft loss, all-cause mortality, or doubling of serum creatinine, without an increased incidence of adverse events.

## Graphical abstract

**Figure:**
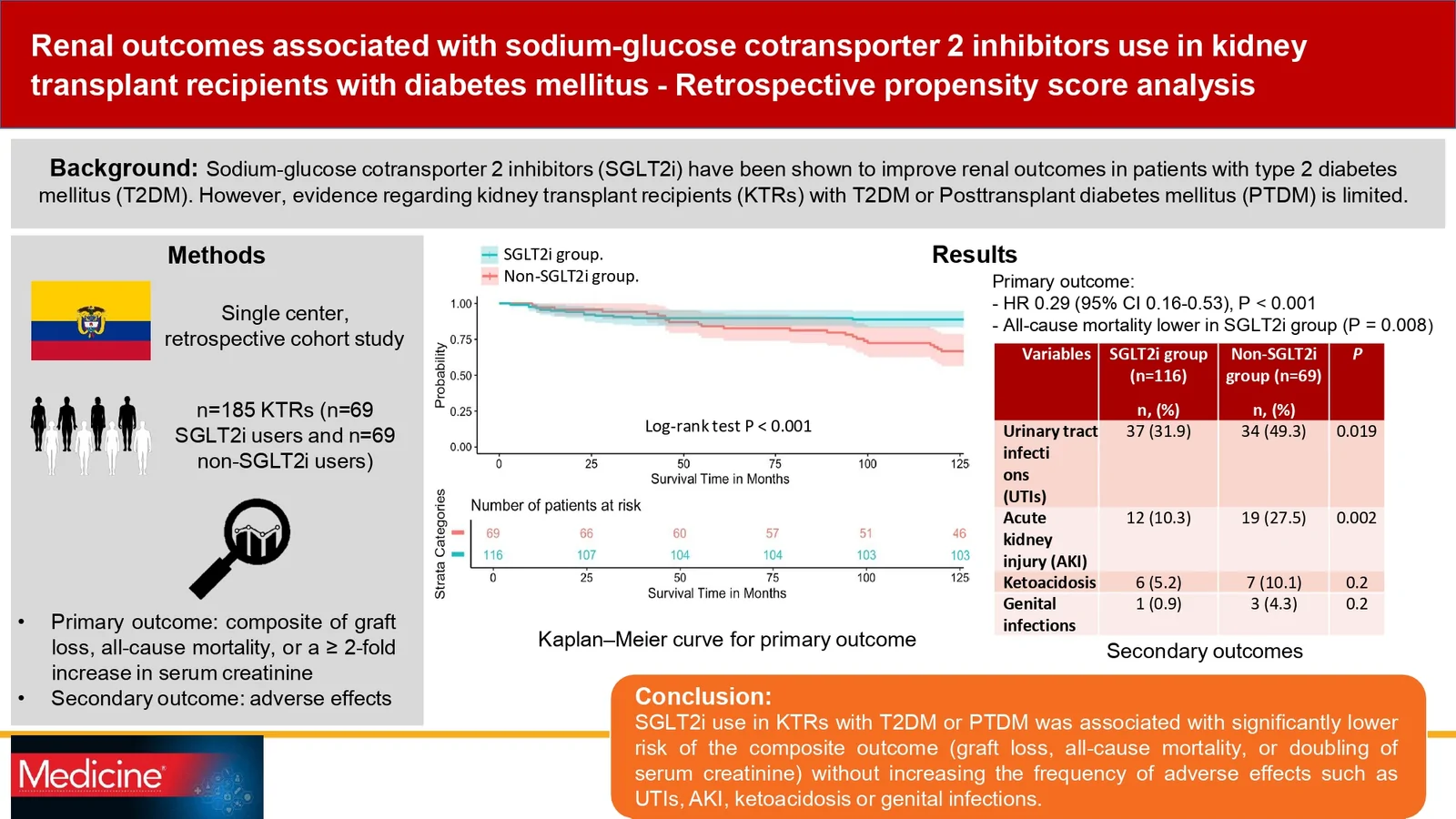

## 1. Introduction

Posttransplant diabetes mellitus (PTDM) markedly worsens post-renal-transplant outcomes. In a meta-analysis of 14 studies including nearly 75,000 kidney transplant recipients, Lim et al demonstrated that the development of PTDM was associated with a 67 % higher risk of all-cause mortality and a 35 % higher risk of graft failure, even after multivariable adjustment for major confounders such as age, comorbidities, and immunosuppressive regimen.^[1]^ DM may preexist as progression to end-stage renal disease (ESRD)-related^[2]^ or develop posttransplant (PTDM).^[3]^ Both DM and PTDM heighten microalbuminuria risk posttransplant,^[4]^ necessitating stringent glycemic control. PTDM shares pathophysiology with type 2 diabetes (T2DM), suggesting analogous oral antidiabetic strategies^[3,5]^

Sodium-glucose cotransporter 2 inhibitors (SGLT2i) are associated with lower HbA1c via renal glucose excretion, with added benefits for weight, blood pressure,^[6]^ and cardiovascular risk reduction in T2DM.^[7]^ Their nephroprotective effects, including intraglomerular pressure reduction,^[8]^ and improvements in albuminuria, kidney function, and renal mortality in T2DM,^[9]^ are evidenced by trials with canagliflozin,^[10]^ dapagliflozin,^[11]^ and empagliflozin.^[12]^

Recent multicenter studies have explored SGLT2i in kidney transplant recipients (KTRs). Sánchez et al (2023) conducted an observational multicenter study (N = 339) demonstrating SGLT2i efficacy in glycemic control, weight reduction, and proteinuria improvement, with urinary tract infections (14%).^[13]^ Lim et al (2022) analyzed 2083 diabetic KTRs and reported an association between SGLT2i use and composite outcomes of mortality, graft failure, and creatinine doubling, with stable long-term eGFR.^[14]^ Their subsequent work (2024) highlighted cardiovascular benefits, including lower major adverse cardiovascular events (MACE) and myocardial infarction rates in a propensity-matched cohort. These studies, however, focused on Asian populations or short-term follow-up, leaving gaps in understanding long-term graft outcomes in diverse cohorts.^[15]^ A recent scoping review published by Mreyoud et al in 2024 examined the efficacy and safety of sodium-glucose cotransporter-2 (SGLT2) inhibitors in solid organ transplant recipients. While this review provides a broad overview of the current experience, the authors themselves concluded that the available evidence remains limited, heterogeneous, and insufficient to support strong recommendations – particularly in specific populations such as kidney transplant recipients with diabetes.^[16]^

We recognized the need for more focused and methodologically robust studies in this area. The absence of strong, population-specific data – particularly in kidney transplant recipients with diabetes – prompted us to undertake the present study. In this context, we aimed to contribute meaningful, real-world evidence by conducting an observational study in a Latin American cohort, a population that remains notably underrepresented in the existing literature. Therefore, the primary objective was to evaluate the clinical response to SGLT2 inhibitor therapy using a clinically relevant composite outcome (graft loss, sustained doubling of serum creatinine, or mortality) in kidney transplant recipients with diabetes. The secondary outcomes included adverse effects related to the treatment. Through this approach, we seek to address key evidence gaps and support informed clinical decision-making regarding the use of SGLT2 inhibitors in this high-risk population.

## 2. Materials and methods

### 2.1. Study design and participants

This retrospective cohort study at Fundación Valle del Lili University Hospital (Cali, Colombia) included adults with kidney transplantation (2011–2022), pretransplant T2DM or PTDM according to American Diabetes Association (ADA) criteria,^[17]^ eGFR ≥20 mL/min/1.73 m^2^ (CKD-EPI 2009),^[18]^ and ≥6-month follow-up. Exclusions included T1DM, combined transplants, or graft loss <12 months. Exposure was defined as SGLT2i use ≥90 days, verified via pharmacologic reconciliation.

### 2.2. Data collection and outcomes

Data from the TRENAL database and medical records were managed via REDCap. The primary outcome was a composite of graft loss, mortality, or serum creatinine ≥2× baseline >3 months. Baseline serum creatinine was defined as the serum creatinine measured at posttransplant week 4; the “doubling of serum creatinine” component was defined as a ≥2-fold increase relative to this week-4 value, occurring >3 months after baseline. Secondary outcomes included individual components, HbA1c/eGFR/UPCR trends, and adverse effects (AKI [KDIGO25], UTIs, genital infections, ketoacidosis).

### 2.3. Statistical analysis

The sample size calculation was based on an interim analysis of the Transplantation and Renal Database (TRENAL). Between 2011 and 2023, 1000 patients were registered, of whom 200 were diagnosed with T2DM. Additionally, an estimated 11% of patients had PTDM according to previous institutional study data,^[19]^ resulting in an expected 300 patients with T2DM or PTDM. A study by Lim et al,^[14]^ which evaluated a composite outcome (all-cause mortality, kidney graft failure, or a ≥ 2-fold increase in creatinine from baseline) occurring in approximately 6% of SGLT2is users compared to approximately 23% of non-SGLT2i users, detecting a hazard ratio (HR) of 0.21 with 80% statistical power and a 95% confidence level, required 67 patients in each group (SGLT2i users and non-SGLT2i users), totaling 134 individuals. Exposure to SGLT2i was modeled as a time-fixed variable.

Categorical variables are presented as proportions and were analyzed using the chi-square test or Fisher exact test for each risk factor. Continuous variables are expressed as medians with interquartile ranges or means with standard deviations, depending on the data distribution assessed by the Shapiro–Wilk test. Nonparametric (Mann–Whitney or Wilcoxon test) or parametric tests (*t* test or ANOVA) were used accordingly. For survival analysis, the time to event (primary outcome) was calculated from the initial date of management initiation, with or without SGLT2i. Censorship was defined as death or loss to follow-up (absence of consultations/follow-up for >6 months). The Kaplan–Meier method was used to estimate the time to event for each treatment group.

**Figure 1.**
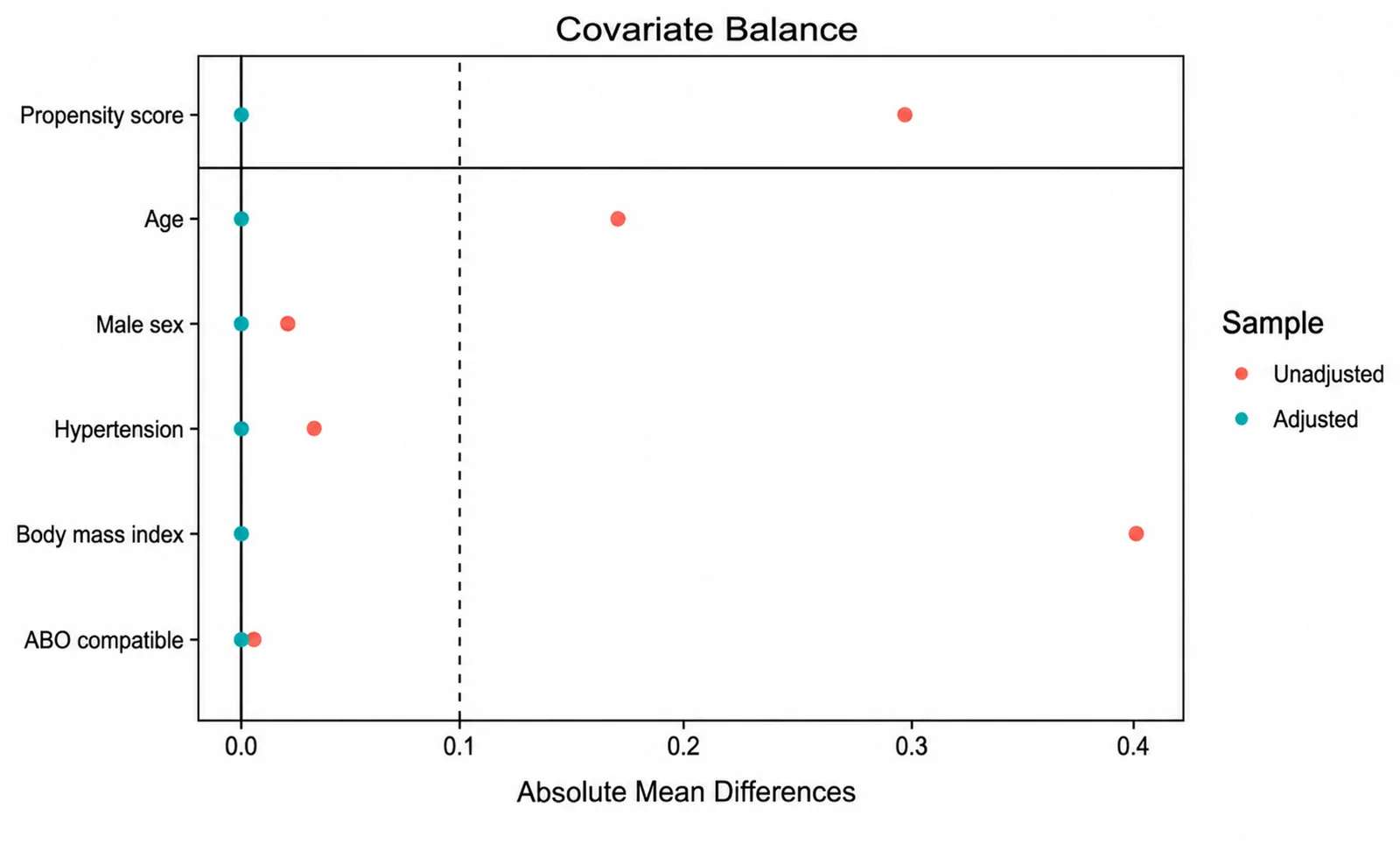
Covariate balance before and after propensity score weighting.

We performed propensity score overlap weighting (overlap weights; PSM) to balance baseline characteristics between patients receiving iSGLT2 inhibitors and controls. Propensity scores, representing the conditional probability of treatment assignment given observed covariates, were estimated using a multivariable logistic regression model. The model included clinically relevant baseline variables: age, sex, body mass index, ABO group compatibility, and hypertension status. Subsequently, overlap weighting was applied to estimate the average treatment effect in the overlap population (ATO), corresponding to patients with the greatest clinical comparability between the exposure groups. Propensity scores were estimated using logistic regression. Covariate balance before and after weighting was evaluated using absolute standardized mean differences with values <0.10 considered indicative of adequate balance (Fig. 1). This adjustment achieved statistical equivalence between the groups in their baseline characteristics, thereby reducing confounding bias. The association between SGLT2 inhibitor use and time-to-event was analyzed using a weighted Cox proportional hazards model incorporating overlap weights and robust sandwich variance estimators. Results were expressed as hazard ratios (HRs) with 95% confidence intervals (95% CIs). Adjusted survival curves by group were derived from the weighted Cox model. The proportional hazards assumption was assessed using Schoenfeld residuals.

All analyses were performed using R Studio software version 4.3.3 (The R Foundation for Statistical Computing, Vienna, Austria, URL https://www.R-project.org/). Statistical significance was set at *P* < .05.

### 2.4. Ethical considerations

This study was approved by the Institutional Ethics Committee (Institutional Review Board) of Fundación Valle del Lili University Hospital (Cali, Colombia; approval number 2023-125). Given the retrospective observational nature of the study and the use of routinely collected clinical data, the committee waived the requirement for individual informed consent.

## 3. Results

### 3.1. Patient characteristics

The flow diagram of participant selection is described in Figure S1, Supplemental Digital Content 1. A total of 278 patients were identified in the TRENAL database during the study period, and 93 were excluded (Fig. S1, Supplemental Digital Content 1). Finally, 185 patients met the eligibility criteria, with an average follow-up time of 121 months.

Table 1 presents the demographic and clinical characteristics estimated using overlap weighting. The real sample size was n = 185; for Table 1 variables, complete-case information was available for N = 83, and the weighted N shown in the table header corresponds to the sum of overlap weights (Overall N = 83; N = 41 per group). The median age was 58.0 years (IQR 52.0–64.0), and 57.3% were male. T2DM was present in 57.7%, and PTDM in 41.9%. Regarding renal replacement therapy prior to transplant, 66.3% received hemodialysis, 28.7% peritoneal dialysis, and 5.0% were pre-dialysis. The median dialysis vintage was 3.0 years (IQR 2.0–6.0; N = 80). Microvascular complications included diabetic retinopathy in 18.3% and diabetic neuropathy in 22.8%. Most patients had no prior cardiovascular disease (82.0%); stroke and peripheral artery disease were present in 7.2% and 7.6%, respectively. No statistically significant differences were observed between groups after overlap weighting across the variables reported in Table 1.

**Table 1:** Baseline clinical and demographic characteristics of the study population according to SGLT2 inhibitor exposure.

	Before overlap propensity score weighting				After overlap propensity score weighting*			
Variables	Total (N = 185)	SGLT2i group (N = 116)	Non‑SGLT2i group (N = 69)	*P*‑value‡	Total (N* = 83)	SGLT2i group (N* = 41)	Non‑SGLT2i group (N* = 41)	*P*‑value‡
Age	57.8 (9.1)†	58.39 (9.0)†	56.81 (9.3)†		58.0 (52.0, 64.0)	58.0 (52.0, 64.0)	57.0 (51.0, 64.0)	.8
Male sex, n (%)	109 (58.9%)	69 (59.5%)	40 (58%)	.840	47 (57.3%)	23 (57.1%)	24 (57.5%)	>.9
Type of renal replacement therapy pre‑transplant, n (%)				.874				.9
Pre‑dialysis	10 (5.5%)	7 (6.1%)	3 (4.3%)		4 (5.0%)	2 (5.2%)	2 (4.7%)	
Peritoneal dialysis	52 (28.4%)	32 (28.1%)	20 (29%)		24 (28.7%)	12 (28.4%)	12 (28.9%)	
Hemodialysis	121 (66.1%)	75 (65.8%)	46 (66.7%)		55 (66.3%)	27 (66.3%)	27 (66.3%)	
Missing data	2	2	0		3	2	1	
Time window on dialysis before transplant	4.32 (3.4)†	4.1 (3)†	4.73 (4)†	.246	3.0 (2.0, 6.0)	3.0 (2.0, 6.0)	3.0 (1.0, 6.0)	.8
Unknown	3	2	1		3	2	1	
Diagnosis of PTDM, n (%)	76 (41.1%)	42 (36.2%)	34 (49.3%)	.08	35 (41.9%)	15 (36.7%)	20 (47.2%)	.2
T2DM, n (%)	109 (58.9%)	74 (63.8%)	35 (50.7%)	.08	48 (57.7%)	26 (62.6%)	22 (52.8%)	.2
Diabetic retinopathy, n (%)	33 (17.8%)	22 (19%)	11 (15.9%)	.603	15 (18.3%)	8 (19.9%)	7 (16.7%)	.6
Diabetic neuropathy, n (%)	42 (22.7%)	26 (22.4%)	16 (23.2%)	.903	19 (22.8%)	8 (20.3%)	10 (25.3%)	.4
No cardiovascular disease, n (%)	32 (17.3%)	22 (19%)	10 (14.5%)	.437	68 (82.0%)	34 (81.9%)	34 (82.2%)	>.9
Stroke, n (%)	11 (5.9%)	4 (3.4%)	7 (10.1%)	.104	6 (7.2%)	1 (3.6%)	4 (10.8%)	.057
Peripheral artery disease, n (%)	17 (9.2%)	14 (12.1%)	3 (4.3%)	.079	6 (7.6%)	4 (10.8%)	2 (4.4%)	.14
Other, n (%)	1	1	1		1 (1.0%)	0 (0.9%)	1 (1.2%)	.8

IQR = interquartile range, PTDM = posttransplant diabetes mellitus, SGLT2 = sodium-glucose cotransporter 2, SGLT2i = sodium-glucose cotransporter 2 inhibitor(s), T2DM = type 2 diabetes mellitus.

* After applying overlap propensity score weighting, a single individual no longer represents a single data point, instead, the weighted N is the sum of the weights, rounded to 1 decimal.

† Mean (standard deviation).

‡ Wilcoxon rank sum test; Pearson Chi-squared test; Fisher exact test, Design-based Kruskal–Wallis test; Pearson χ^2^: Rao & Scott adjustment.

Post-weighting analysis demonstrated similar distributions of baseline characteristics between the SGLT2 inhibitor and non-SGLT2 inhibitor groups, supporting adequate balance.

### 3.2. Transplant characteristics and treatment

Most patients received hemodialysis before transplantation (66.3%). Sixty-three percent had 3 to 4 incompatible HLA alleles, and 80% had a PRA ≤30%. The most frequent cold ischemia time ranged from 11to 18 hours.

Pharmacological treatment and early posttransplant parameters are detailed in Table 2. Metformin was used in 62.8% of patients, sulfonylureas in 1.8%, and GLP-1 receptor agonists in 3.8%. Use of DPP4 inhibitors differed between groups, being more frequent in the SGLT2i group (46.4%) than in the non-SGLT2i group (27.0%; *P* = .011). HbA1c at 3 to 6 months posttransplant was similar between groups (median 7.2% overall; *P* > .9; N = 58). Tacrolimus use was high among patients with available data (N = 75), with no differences between groups (*P* = .7). Regarding calcineurin inhibitor therapeutic exposure during the first posttransplant year (N = 83), 44.2% were always within the optimal range (100% of the time), and 21.9% were within range 75% to 99% of the time; distributions did not differ between groups (*P* = .5). Serum creatinine at 1 to 3 months posttransplant was 1.3 (IQR 1.0–1.6) overall and did not significantly differ between groups (*P* = .067; N = 83).

**Table 2:** Pharmacological treatment and early posttransplant according to SGLT2 inhibitor exposure.

	Before overlap propensity score weighting				After overlap propensity score weighting*			
Variables	Total (N = 185)	SGLT2i group (N = 116)	Non‑SGLT2i group (N = 69)	*P*‑value†	Total (N* = 83)	SGLT2i group (N* = 41)	Non‑SGLT2i group (N* = 41)	*P*‑value†
Metformin, n (%)	118 (64%)	76 (66%)	42 (61%)	.5	52 (62.8%)	27 (65.7%)	25 (59.9%)	.4
Sulfonylurea, n (%)	4 (2.2%)	3 (2.6%)	1 (1.4%)	>.9	2 (1.8%)	1 (2.4%)	1 (1.2%)	.6
DPP4‑i, n (%)	75 (41%)	55 (48%)	20 (29%)	.012	30 (36.6%)	19 (46.4%)	11 (27.0%)	.011
Unknown	1	1	0		–	–	–	
GLP‑1A, n (%)	8 (4.3%)	6 (5.2%)	2 (2.9%)	.7	3 (3.8%)	2 (4.4%)	1 (3.1%)	.7
HbA1c 3–6 mo (%), (n=)	7.24 (6.40, 8.65)	7.30 (6.30, 8.90)	7.20 (6.40, 8.50)	>.9	7.2 (6.4, 8.6)	7.3 (6.3, 8.9)	7.2 (6.4, 8.5)	>.9
Unknown	57	39	18		25	14	11	
Tacrolimus, n (%)	166 (98%)	108 (97%)	58 (98%)	>.9	73 (97.7%)	39 (97.4%)	34 (98.1%)	.7
Unknown	15	5	10		0 (0.5%)	0 (1.0%)	0 (0.0%)	
Cyclosporine, n (%)	28 (100%)	9 (100%)	19 (100%)		15 (100.0%)	3 (100.0%)	12 (100.0%)	–
Unknown	157	107	50		68 (81.9%)	37 (90.2%)	32 (76.2%)	
Initial optimal levels of CNIs, n (%)				.4				.5
Always (100%)	81 (44%)	52 (45%)	29 (42%)		37 (44.2%)	17 (41.0%)	20 (47.0%)	
Almost always (75–99%)	43 (23%)	29 (25%)	14 (20%)		18 (21.9%)	9 (22.3%)	9 (21.5%)	
Often (50–74%)	32 (17%)	16 (14%)	16 (23%)		15 (18.4%)	10 (24.3%)	5 (11.5%)	
Rarely (<50%)	28 (15%)	18 (16%)	10 (14%)		13 (15.4%)	5 (12.4%)	8 (20.0%)	
Nephrotoxicity, n (%)	8 (80%)	4 (80%)	4 (80%)		4 (87.4%)	3 (83.0%)	1 (100.0%)	.7
Unknown	175	111	64		78 (94.0%)*	39 (95.1%)*	39 (92.9%)*	
Tobacco use, n (%)				.5				.3
Never	101 (55%)	60 (52%)	41 (59%)		47 (86.3%)	21 (85.3%)	26 (87.3%)	
Former smoker	17 (9.2%)	13 (11%)	4 (5.8%)		7 (13.1%)	4 (14.1%)	3 (10.4%)	
Missing data	66 (36%)	42 (36%)	24 (35%)		28	14	14	
Serum creatinine at 1–3 mo post‑transplant	1.27 (0.98, 1.60)	1.22 (0.94, 1.56)	1.31 (1.09, 1.62)	.10	1.3 (1.0, 1.6)	1.2 (0.9, 1.5)	1.3 (1.1, 1.6)	.067
Composite outcome				.8				<.001
Death	26 (14%)	9 (7.8%)	17 (25%)	.001	14 (17.0%)	3 (7.9%)	11 (26.0%)	.001
Graft loss	12 (6.5%)	5 (4.3%)	7 (10%)	.13	6 (7.6%)	2 (5.2%)	4 (10.0%)	.2
≥2‑fold increase in serum creatinine from baseline	15 (8.1%)	8 (6.9%)	7 (10%)	.4	7 (9.0%)	3 (7.5%)	4 (10.4%)	.5

CNI = calcineurin inhibitor, DPP4-i = dipeptidyl peptidase-4 inhibitor, IQR = interquartile range, SGLT2 = sodium-glucose cotransporter 2, SGLT2i = sodium-glucose cotransporter 2 inhibitor(s).

* After applying overlap propensity score weighting, a single individual no longer represents a single data point, instead, the weighted N is the sum of the weights, rounded to 1 decimal.

† Wilcoxon rank sum test; Pearson Chi-squared test; Fisher exact test, Design-based Kruskal–Wallis test; Pearson χ^2^: Rao & Scott adjustment.

### 3.3. Outcome

Event rates for the composite primary outcome and its individual components are summarized in Table 2. The composite outcome occurred less frequently in the SGLT2i group (16.9%) compared to the non-SGLT2i group (47.8%; *P* < .001). All-cause mortality was also lower in the SGLT2i group (7.9% vs 26.0%, *P* = .001). No statistically significant differences were observed for graft loss (5.2% vs 10.0%, *P* = .2) or for a ≥2-fold increase in serum creatinine from baseline (7.5% vs 10.4%, *P* = .5).

**Figure 2.**
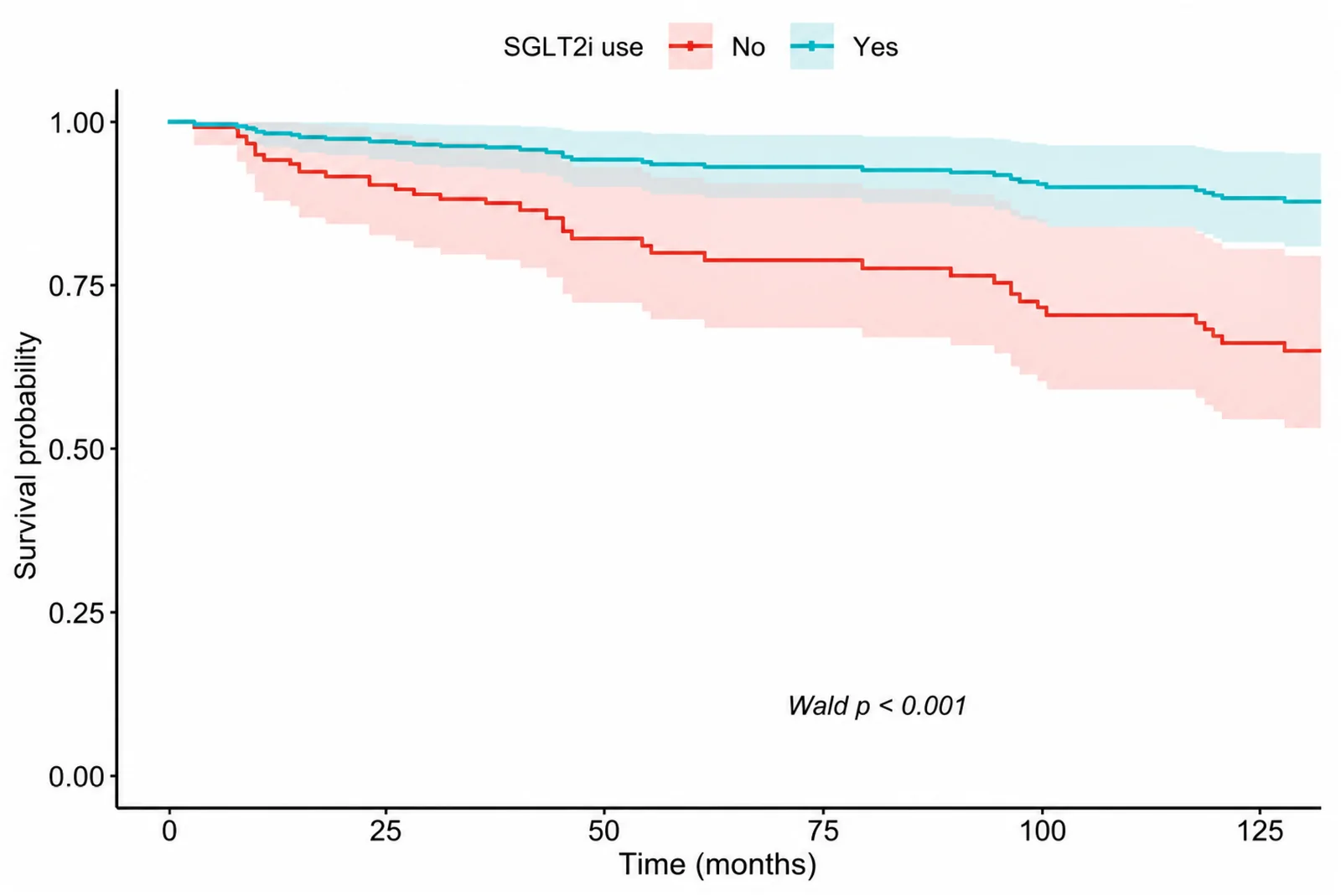
Kaplan–Meier after propensity score weighting for the composite primary outcome. SGLT2i = sodium-glucose cotransporter 2 inhibitor(s).

In an overlap-weighted Cox regression model, SGLT2i use was associated with a lower hazard of the composite endpoint (HR 0.30, 95% CI: 0.17–0.55, *P* < .001), corresponding to an approximately 70% lower hazard compared with overlap-weighted controls (Fig. 2).

### 3.4. Adverse effects

Adverse events available in the overlap-weighted outputs are summarized in Table 3 (N varies by outcome due to missingness). Urinary sepsis/hospitalization categories did not differ between groups (*P* = .2; N = 83). Among patients with recorded data for the number of urinary tract infection episodes (N = 45), the distribution of episode counts showed no statistically significant difference between groups (*P* = .076). Ketoacidosis occurred in 7.9% of patients and genital infections in 2.7%, with no significant differences between groups (both *P* = .3; N = 83).

**Table 3:** Adverse effects according to SGLT2 inhibitor exposure after propensity score weighting.

	After overlap propensity score weighting*			
Variables	Total (N* = 83)	SGLT2i group (N* = 41)	Non‑SGLT2i group (N* = 41)	*P*‑value†
UTIs, n (%)	0 (0.5%)	0 (0.9%)	0 (0.0%)	–
Sepsis or hospitalization due to UTIs, n (%)	24 (29.0%)	10 (23.0%)	15 (35.0%)	.2
AKI (not associated with rejection), n (%)	14 (63.8%)	5 (61.2%)	8 (65.3%)	.8
Ketoacidosis, n (%)	7 (7.9%)	2 (5.7%)	4 (10.1%)	.3
Genital infection, n (%)	2 (2.7%)	1 (1.3%)	2 (4.1%)	.3

AKI = acute kidney injury, IQR = interquartile range, SGLT2 = sodium-glucose cotransporter 2, SGLT2i = sodium-glucose cotransporter 2 inhibitor(s), UTIs = urinary tract infections.

* After applying overlap propensity score weighting, a single individual no longer represents a single data point; instead, the weighted N is the sum of the weights, rounded to 1 decimal.

† Design-based Kruskal–Wallis test; Pearson χ^2^: Rao & Scott adjustment.

## 4. Discussion

Given the non-randomized nature of treatment assignment in our cohort, we anticipated systematic differences in baseline characteristics between patients receiving SGLT2 inhibitors and controls. To address potential confounding bias, we performed propensity score overlap weighting (overlap weights) to balance baseline covariates. This study retrospectively evaluated the effect and safety of SGLT2i on renal outcomes in KTRs with T2DM or PTDM. The main finding was that the primary composite outcome, which included graft loss, all-cause mortality, and doubling of serum creatinine from baseline, was lower in the SGLT2i group than in the non-SGLT2i group. This association persisted after overlap weighting, suggesting it was not explained by measured baseline covariate imbalances between groups. Additionally, SGLT2i was found to be safe in this patient group, with no significant differences in urinary sepsis/hospitalization categories, the distribution of urinary tract infection episode counts (among those with available data), genital infections, or ketoacidosis.

The significant reduction in all-cause mortality observed in the SGLT2i group, despite well-balanced baseline characteristics including age, cardiovascular risk, and renal function after overlap-weighted analysis, merits further consideration. This finding suggests that the survival benefit may be directly linked to the specific pharmacological effects of SGLT2 inhibitors, rather than being solely attributable to differences in patient profiles. This phenomenon aligns with the results of the landmark EMPA-REG OUTCOME trial, which demonstrated a significant reduction in cardiovascular and all-cause mortality with empagliflozin in patients with type 2 diabetes and established cardiovascular disease, an effect that emerged early and was consistent across subgroups.^[20]^The mechanisms underlying this mortality benefit are thought to be multifactorial, potentially involving positive effects on hemodynamics, vascular function, cardiac load, and energy metabolism, which are not fully captured by conventional risk factors. While our study was not powered to delineate the precise mechanisms in a kidney transplant population, the concordance of our mortality finding with this pivotal trial strengthens the hypothesis that SGLT2 inhibitors confer a direct survival advantage that extends to high-risk populations such as KTRs.

The 90-day threshold for SGLT2i exposure was based on prior evidence demonstrating time-dependent renoprotective effects. Chen et al, in a cohort of 21,947 patients, observed that the beneficial effects of SGLT2 inhibitors first achieved statistical significance at 26 days of treatment.^[21]^ Furthermore, Huang et al demonstrated that SGLT2i exposure exceeding 90 days was associated with significant reductions in the risk of dialysis initiation and renal failure.^[22]^ To date, only 5 studies have similarly evaluated the use of SGLT2i compared to a non-exposed group.^[15,23-26]^ Two of these studies specifically assessed renal^[14]^ and cardiovascular outcomes.^[15]^In 2022, Lim et al^[25]^ conducted a retrospective cohort study on 202 SGLT2i users compared to 554 non-SGLT2i users, using propensity score adjustment to evaluate a primary outcome similar to ours. Similarly, they also found significant differences favoring the use of SGLT2i for the primary composite outcome. In their study, the individual components of the outcome remained significant, which only occurred for all-cause mortality in our study (Table 2). We believe that despite the lower percentage in the SGLT2i group, the low number of events prevented significant differences in graft loss and doubling of baseline creatinine (Table 2). Similar to Lim et al,^[25]^ we found that the protective effect of SGLT2i on the primary outcome persisted in the overlap-weighted Cox regression model (Fig. 2).

Urinary infectious complications are common in kidney transplant recipients, with prevalences around 35% reported in a large meta-analysis of 72,600 patients.^[13,14,23-25,27-29]^ In our cohort, urinary sepsis/hospitalization categories did not differ between groups (Table 3), and the distribution of urinary tract infection episode counts among patients with available data was also not significantly different. These findings differ from those of most studies with comparison groups, where UTI frequency was similar between the SGLT2i group and the non-SGLT2i group (*P* > .05).^[21-25]^ Similarly, Lim et al reported no differences in UTI incidence between SGLT2i users, with 4.5 events/100 patient-years, and non-SGLT2i users, with 6.2 events/100 patient-years; the RR was 0.74 (95% CI: 0.42–1.31). Schwaiger et al^[19]^ also found no differences in UTI incidence when comparing empagliflozin use with a reference nonexposed population (KTRs and PTDM; *P* = .81). Previous UTIs are a risk factor for their development, with an OR of 1.31 (95% CI: 1.05–1.63), a risk also seen in SGLT2i users, with an OR of 7.9 (3.63–17.21), being more common in women, with an OR of 2.46 (95% CI: 1.19–5.03).^[13]^ Considering these factors, our results may be biased because treating physicians are likely to avoid SGLT2i use in higher-risk UTI patients.

Initiating treatment with SGLT2i often results in an initial decrease in the eGFR over several weeks, which usually recovers and stabilizes.^[30,31]^ Given the known early eGFR dip after SGLT2i initiation, AKI remains a relevant safety concern.^[28-31]^ In our overlap-weighted analysis, AKI not associated with rejection did not differ significantly between groups (Table 3). Serum creatinine measurements were performed in the institutional reference laboratory, which undergoes regular quality control studies. Fundacion Valle del Lili ranks #1 in Colombia,^[32]^ with consistent quality assurance protocols. In a review of various studies, the frequency of AKI was not specifically reported^[14,23-25,28]^ or was lower than that in our study (1.8–10%).^[13,29]^ Sánchez et al^[18]^ reported that AKI occurred more frequently in patients with a baseline eGFR < 40 mL/min/1.73 m^2^ than in those with a baseline eGFR ≥ 40 mL/min/1.73 m^2^ (*P* = .003). Other studies, though not reporting the frequency of AKI, found that the eGFR significantly decreased between weeks 4 and 8 after SGLT2i administration; however, in all patients, the eGFR recovered within 6 to 12 months, with no significant differences between SGLT2i and non-SGLT2i users.^[14,23,28]^ Consistent with our findings, 2 meta-analyses concluded that SGLT2i use in non-transplanted T2DM patients is associated with a reduced AKI risk by 36%^[33]^ and 41%.^[34]^ However, our findings may be biased for 2 reasons: physician profiling of lower-risk AKI patients and the fact that the non-SGLT2i group had a shorter transplant/antidiabetic window than did exposed patients (Table 1), with this organ being more susceptible to AKI during the first months posttransplant. In addition, the retrospective and non-randomized design may have led to selection bias in treatment allocation, which could have influenced observed safety outcomes.

Finally, we observed other adverse effects, such as ketoacidosis and genital infections, in 7.9% and 2.7% of patients, respectively, with no significant differences between the SGLT2i and non-SGLT2i groups (Table 3). Similarly, both conditions were infrequently reported in most other studies (0–7%),^[13,23,25,27,29,35,36]^ and in most cases, this did not limit SGLT2i use in this population.

This study is noteworthy because it is the fourth-largest cohort published with the greatest number of KTRs with T2DM or PTDM treated with SGLT2i (n = 116), following the work of Sánchez et al,^[18]^ Lim et al,^[24]^ and Lim et al^[25]^ with 339, 202, and 127 patients, respectively. This study has several limitations. First, it was conducted at a single institution, favoring selection and single-center bias. Second, this retrospective study has information bias, although this bias was mitigated by obtaining data from the most direct sources possible. Importantly, albuminuria – a well-established renal prognosis marker – could not be analyzed longitudinally due to a lack of periodic measurements attributable to health system limitations. This restricted our ability to evaluate its role as a treatment target with SGLT2i. Third, primary outcome results are subject to confounding factors inherent to observational design; therefore, we used a propensity score overlap weighting approach and an overlap-weighted Cox regression model to reduce confounding. Nevertheless, the limited number of outcome events may have reduced the precision of the estimated associations and increased the risk of type II error. This limitation is commonly discussed in relation to the events-per-variable framework proposed by Peduzzi^[37]^; and more flexible criteria suggested by other authors.^[37,38]^ Nevertheless, the low number of outcome events limited the statistical power of the multivariate analysis and may have reduced the precision of the estimated associations, increasing the risk of type II error. Additionally, a potential temporal bias between cohorts should be acknowledged, as control patients were transplanted and managed in earlier periods, possibly reflecting differences in immunosuppressive protocols, follow-up intensity, and overall posttransplant care.

The observed difference in the interval between transplantation and the initiation of antidiabetic therapy represents a noteworthy finding (Table 1). Although this study was not specifically designed to explore the underlying causes of this disparity, it may be attributable to clinical inertia in the intensification of antihyperglycemic management within the posttransplant population. Furthermore, as this value represents a mean, it is influenced by the entire distribution, including patients with both very short and very long treatment initiation windows; the presence of extreme values in either group could therefore skew the average. The availability of SGLT2i may play an important role in this difference. Other researchers report a mean time between transplant date and initiation of therapy of 28 months (IQR = 11–91), which is similar to our findings,^[37,39]^ At this point, it is important to consider that patients receiving SGLT2i treatment may represent more recent transplant recipients. Pharmacological adherence could not be objectively assessed, although it was verified in the medical records and prescriptions at each medical consultation for continuous medication use.

## 5. Conclusions

This study suggested that SGLT2i in KTRs with T2DM or PTDM was associated with nephroprotective effects reflected in a lower combined risk of graft loss, all-cause mortality, or doubling the baseline creatinine level. Additionally, they have been shown to be safe in this special population, without increasing the frequency of adverse effects, including urinary sepsis/hospitalization, AKI not associated with rejection, ketoacidosis, and genital infections. However, the observational nature of this study only suggests hypotheses that need to be confirmed through randomized clinical trials with long-term follow-up. Currently, several such studies are registered at ClinicalTrials.gov (NCT05390892, NCT04906213, and NCT04743453).

## Author contributions

**Conceptualization:** Guillermo Edinson Guzmán, Fabio Figueroa, Santiago Granados, Oriana Arias-Valderrama, Carlos Eduardo Durán.

**Investigation:** Oriana Arias-Valderrama.

**Methodology:** Oriana Arias-Valderrama.

**Project administration:** Guillermo Edinson Guzmán.

**Software:** Oriana Arias-Valderrama.

**Supervision:** Guillermo Edinson Guzmán, Oriana Arias-Valderrama.

**Validation:** Guillermo Edinson Guzmán.

**Writing – original draft:** Guillermo Edinson Guzmán, Oriana Arias-Valderrama, Juan Lukas Ordóñez, Carlos Eduardo Durán.

**Writing – review & editing:** Guillermo Edinson Guzmán, Oriana Arias-Valderrama, Juan Lukas Ordóñez.



## References

[1] Lin H Yan J Yuan L. Impact of diabetes mellitus developing after kidney transplantation on patient mortality and graft survival: a meta-analysis of adjusted data. Diabetol Metabol Syndrome. 2021;13:126. 10.1186/s13098-021-00742-4 PMC8557540 34717725

[2] Koye DN Magliano DJ Nelson RG Pavkov ME. The global epidemiology of diabetes and kidney disease. Adv Chronic Kidney Dis. 2018;25:121–32. 29580576 10.1053/j.ackd.2017.10.011 PMC11000253

[3] Jenssen T Hartmann A. Post-transplant diabetes mellitus in patients with solid organ transplants. Nat Rev Endocrinol. 2019;15:172–88. 30622369 10.1038/s41574-018-0137-7

[4] Knoll GA. NARRATIVE REVIEW proteinuria in kidney transplant recipients: prevalence, prognosis, and evidence-based management. Am J Kidney Dis. 2009;54:1131–44. 10.1053/j.ajkd.2009.06.031 19726115

[5] Sharif A Chakkera H de Vries APJ. International consensus on post-transplantation diabetes mellitus. Nephrol Dial Transplant. 2024;39:531–49. 38171510 10.1093/ndt/gfad258 PMC11024828

[6] Lupsa BC Inzucchi SE. Use of SGLT2 inhibitors in type 2 diabetes: weighing the risks and benefits. Diabetologia. 2018;61:2118–25. 30132031 10.1007/s00125-018-4663-6

[7] American Diabetes Association Professional Practice Committee. 9. pharmacologic approaches to glycemic treatment: standards of care in diabetes—2024. Diabetes Care. 2024;47:S158–78. 38078590 10.2337/dc24-S009 PMC10725810

[8] Bailey CJ Day C Bellary S. Renal protection with SGLT2 inhibitors: effects in acute and chronic kidney disease. Curr Diab Rep. 2022;22:39–52. 35113333 10.1007/s11892-021-01442-z PMC8888485

[9] Diabetes Work Group. KDIGO 2022 clinical practice guideline for diabetes management in chronic kidney disease kidney I N T E R N A T I O N A L. www.kidney-international.org. Accessed February 8, 2026.

[10] Perkovic V Jardine MJ Neal B; CREDENCE Trial Investigators. Canagliflozin and renal outcomes in type 2 diabetes and nephropathy. N Engl J Med. 2019;380:2295–306. 30990260 10.1056/NEJMoa1811744

[11] Heerspink HJL Stefánsson BV Correa-Rotter R; DAPA-CKD Trial Committees and Investigators. Dapagliflozin in patients with chronic kidney disease. N Engl J Med. 2020;383:1436–46. 32970396 10.1056/NEJMoa2024816

[12] The EMPA-KIDNEY Collaborative Group. Empagliflozin in patients with chronic kidney disease. New Eng J Med. 2023;388:117–27. 36331190 10.1056/NEJMoa2204233 PMC7614055

[13] Shah M Virani Z Rajput P Shah B. Efficacy and Safety of Canagliflozin in Kidney Transplant Patients. Indian J Nephrol. 2019;29:278–81. 31423063 10.4103/ijn.IJN_2_18 PMC6668319

[14] Strøm Halden TA Kvitne KE Midtvedt K. Efficacy and safety of empagliflozin in renal transplant recipients with posttransplant diabetes mellitus. Diabetes Care. 2019;42:1067–74. 30862658 10.2337/dc19-0093

[15] Song CC Brown A Winstead R. Early initiation of sodium-glucose linked transporter inhibitors (SGLT-2i) and associated metabolic and electrolyte outcomes in diabetic kidney transplant recipients. Endocrinol Diabetes Metab. 2021;4:e00185. 33855198 10.1002/edm2.185 PMC8029504

[16] Hisadome Y Mei T Noguchi H. Safety and efficacy of sodium-glucose cotransporter 2 inhibitors in kidney transplant recipients with pretransplant type 2 diabetes mellitus: a retrospective, single-center, inverse probability of treatment weighting analysis of 85 transplant patients. Transplant Direct. 2021;7:e772. 34646935 10.1097/TXD.0000000000001228 PMC8500668

[17] Lemke A Brokmeier HM Leung SB. Sodium-glucose cotransporter 2 inhibitors for treatment of diabetes mellitus after kidney transplantation. Clin Transplant. 2022;36:e14718. 35593882 10.1111/ctr.14718

[18] Sánchez Fructuoso AI Raba AB Deras EB. Sodium-glucose cotransporter-2 inhibitor therapy in kidney transplant patients with type 2 or post-transplant diabetes: an observational multicentre study. Clin Kidney J. 2023;16:1022–34. 37260993 10.1093/ckj/sfad007 PMC10229265

[19] Schwaiger E Burghart L Signorini L. Empagliflozin in posttransplantation diabetes mellitus: a prospective, interventional pilot study on glucose metabolism, fluid volume, and patient safety. Am J Transplant. 2019;19:907–19. 30585690 10.1111/ajt.15223 PMC6590167

[20] Zinman B Wanner C Lachin JM; EMPA-REG OUTCOME Investigators. Empagliflozin, cardiovascular outcomes, and mortality in type 2 diabetes. N Engl J Med. 2015;373:2117–28. 26378978 10.1056/NEJMoa1504720

[21] Chen KY Nie Z Shi R. Time to benefit of sodium-glucose cotransporter-2 inhibitors among patients with heart failure. JAMA Netw Open. 2023;6:e2330754. 37615988 10.1001/jamanetworkopen.2023.30754 PMC10450563

[22] Huang B Yen CL Wu CY. SGLT2 inhibitors reduce the risk of renal failure in CKD stage 5 patients with Type 2 DM. Sci Rep. 2025;15:5872. 39966427 10.1038/s41598-024-81973-z PMC11836049

[23] Mahling M Schork A Nadalin S Fritsche A Heyne N Guthoff M. Sodium-glucose cotransporter 2 (SGLT2) inhibition in kidney transplant recipients with diabetes mellitus. Kidney Blood Press Res. 2019;44:984–92. 31437852 10.1159/000501854

[24] Lim JH Kwon S Jeon Y. The efficacy and safety of SGLT2 inhibitor in diabetic kidney transplant recipients. Transplantation. 2022;106:e404–12. 35768908 10.1097/TP.0000000000004228

[25] Lim JH Kwon S Seo YJ. Cardioprotective effect of SGLT2 inhibitor in diabetic kidney transplant recipients: a multicenter propensity score matched study. Kidney Int Rep. 2024;9:2474–83. 39156155 10.1016/j.ekir.2024.05.022 PMC11328785

[26] American Diabetes Association Professional Practice Committee 2. Classification and diagnosis of diabetes: standards of medical care in diabetes—2022. Diabetes Care. 2022;45:S17–38. 34964875 10.2337/dc22-S002

[27] Levey AS Stevens LA Schmid CH Zhang Y Castro AF III Feldman HI. Authors Current Mailing address: 8. Paul Eggers Kidney & Urology Branch, NIDDK, National Institutes of Health, 6707 Democracy Blvd. 2081.

[28] Khwaja A. KDIGO clinical practice guidelines for acute kidney injury. Nephron Clin Pract. 2012;120:c179–84. 22890468 10.1159/000339789

[29] Guzmán GE Durán CE Manzi E. Diabetes de Novo Post trasplante: 20 años de experiencia en un centro de América Latina. Diálisis y Trasplante. 2018;39:7–14.

[30] Demir ME Özler TE Merhametsiz O. The results of SGLT-2 inhibitors use in kidney transplantation: 1-year experiences from two centers. Int Urol Nephrol. 2023;55:2989–99. 37289399 10.1007/s11255-023-03645-7 PMC10248967

[31] Hosseinpour M Pezeshgi A Mahdiabadi MZ Sabzghabaei F Hajishah H Mahdavynia S. Prevalence and risk factors of urinary tract infection in kidney recipients: a meta-analysis study. BMC Nephrol. 2023;24:284. 37759155 10.1186/s12882-023-03338-4 PMC10523791

[32] Newsweek. Newsweek. World’s Best Hospitals 2024 – Colombia. Newsweek Rankings. Newsweek. 2024.

[33] Chewcharat A Prasitlumkum N Thongprayoon C. Efficacy and safety of SGLT-2 inhibitors for treatment of diabetes mellitus among kidney transplant patients: a systematic review and meta-analysis. Med Sci (Basel). 2020;8:47. 33213078 10.3390/medsci8040047 PMC7712903

[34] DeFronzo RA Reeves WB Awad AS. Pathophysiology of diabetic kidney disease: impact of SGLT2 inhibitors. Nat Rev Nephrol. 2021;17:319–34. 33547417 10.1038/s41581-021-00393-8

[35] van Bommel EJM Muskiet MHA Tonneijck L Kramer MHH Nieuwdorp M van Raalte DH. SGLT2 inhibition in the diabetic kidney—from mechanisms to clinical outcome. Clin J Am Soc Nephrol. 2017;12:700–10. 28254770 10.2215/CJN.06080616 PMC5383382

[36] Menne J Dumann E Haller H Schmidt BMW. Acute kidney injury and adverse renal events in patients receiving SGLT2-inhibitors: a systematic review and meta-analysis. PLoS Med. 2019;16:e1002983. 31815931 10.1371/journal.pmed.1002983 PMC6901179

[37] Peduzzi P Concato J Kemper E Holford TR Feinstein AR. A simulation study of the number of events per variable in logistic regression analysis. J Clin Epidemiol. 1996;49:1373–9. 8970487 10.1016/s0895-4356(96)00236-3

[38] Vittinghoff E McCulloch CE. Relaxing the rule of ten events per variable in logistic and Cox regression. Am J Epidemiol. 2007;165:710–8. 17182981 10.1093/aje/kwk052

[39] Yugueros González A Kanter J Sancho A. Institutional experience with new antidiabetic drugs in kidney transplant. Transplant Proc. 2021;53:2678–80. 34615601 10.1016/j.transproceed.2021.08.042

