# Supplementary material for: Renal outcomes associated with sodium–glucose cotransporter 2 inhibitor use in kidney transplant recipients with type 2 or posttransplant diabetes: A propensity score overlap weighting retrospective cohort study
Source: Medicine (Baltimore). 2026 Sep 4;105(36):e50385. doi: 10.1097/MD.0000000000050385 (PMC13549577; doi:10.1097/MD.0000000000050385)

Supplementary Figure 1. Flow diagram of patients in the study, including those excluded.

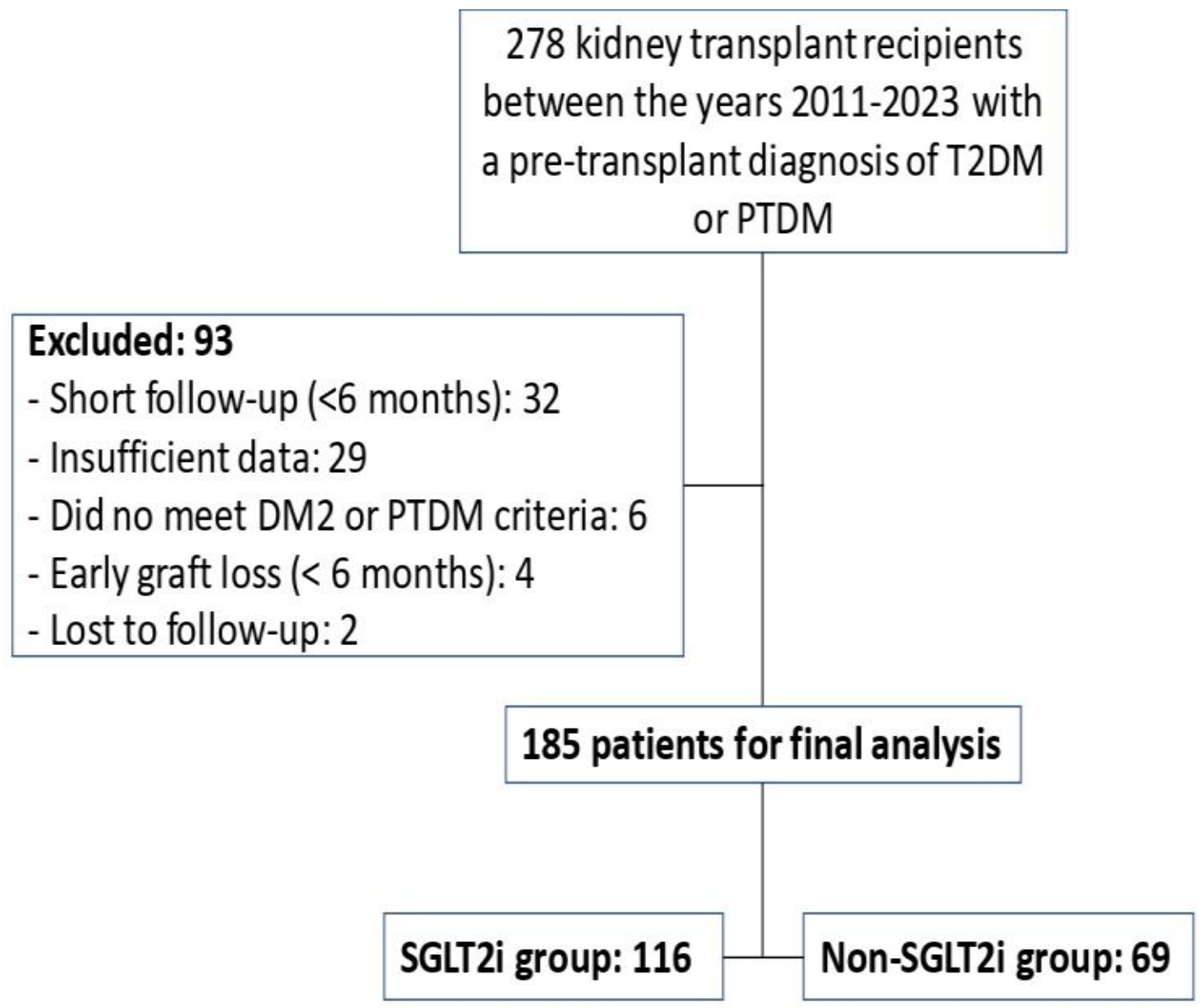

Supplement: Supplementary file 1 [file medi-105-e50385-s001.pdf]
